# New Trends in Supercritical Fluid Technology and Pressurized Liquids for the Extraction and Recovery of Bioactive Compounds from Agro-Industrial and Marine Food Waste

**DOI:** 10.3390/molecules28114421

**Published:** 2023-05-29

**Authors:** Horacio Fraguela-Meissimilly, José Miguel Bastías-Monte, Claudia Vergara, Jaime Ortiz-Viedma, Roberto Lemus-Mondaca, Marcos Flores, Pamela Toledo-Merma, Sylvia Alcázar-Alay, Manuela Gallón-Bedoya

**Affiliations:** 1 Department of Food Engineering, University of Bío-Bío, Av. Andrés Bello 720, Chillán 8370003, Chile; horacio.fraguela@gmail.com; 2 Departamento de Ciencias Básicas, Facultad de Ciencias, Universidad Santo Tomás, Ejercito 146, Santiago 8370003, Chile; 3 Department of Food Science and Chemical Technology, University of Chile, Av. Doctor Carlos Lorca 964, Independencia, Santiago 8380494, Chile; rlemus@uchile.cl (R.L.-M.);; 4 Departamento de Horticultura, Facultad de Ciencias Agrarias, Universidad de Talca, Av. Lircay s/n, Talca 3460000, Chile; 5 Jorge Basadre Grohmann National University, Tacna 23000, Peru; 6 Department of Agricultural and Food Engineering, National University of Colombia, Carrera 65 No. 59A-110, Medellín 4309000, Colombia

**Keywords:** bioactive compounds, food waste, clean extraction, SFE, PLE, patented inventions

## Abstract

Growing consumer interest in healthy foods has led to an increased demand for bioactive compounds derived from eco-technologies. This review highlighted two emerging technologies, pressurized liquid extraction (PLE) and supercritical fluid extraction (SFE), which are based on clean processes aimed at recovering bioactive compounds from different food sources. We studied how the different processing conditions provide many advantages and a great opportunity to obtain compounds with antioxidant, antibacterial, antiviral, or antifungal activity from plant matrices and industrial biowaste, especially antioxidant compounds (anthocyanins and polyphenols) due to their important role in health promotion. Our research was conducted through a systematic search in different scientific databases related to the PLE and SFE topics. The review analyzed the optimal extraction conditions using these technologies, which lead to the efficient extraction of bioactive compounds, the use of different equipment, and recent combinations of SFE and PLE with other emerging technologies. This has given rise to the development of new technological innovations, new commercial applications, and the detailed recovery of various bioactive compounds extracted from different plant and marine life food matrices. These two environmentally friendly methodologies are fully valid and have great future application prospects in biowaste valorization. They represent a feasible technological tool that can promote the implementation of a circular economy model for the food industry. The underlying mechanisms of these techniques were discussed in detail and supported by current literature.

## 1. Introduction

The various industrial processes involved in food production for mass consumption generate large volumes of waste (so-called byproducts) that are usually disposed of and cause serious environmental pollution. This situation has provided an incentive for the industry and researchers to make great efforts to valorize waste biomass by developing technologies that can extract efficiently and cleanly its bioactive components. Agro-industrial waste includes the residues of natural materials derived from processing such as husks, skins, hides, stalks, leftover pulp, and other plant materials [1,2]. Although these agro-industrial byproducts are mainly composed of polysaccharides and fiber, which represent up to 90% of dry weight, they have other components with high bioactive value and health benefits such as polyphenols, flavonoids, lipids, carotenoid pigments, tocopherols, and sterols. Therefore, they have become increasingly popular as functional food ingredients [2].

Pressurized liquid extraction (PLE) and supercritical fluid extraction (SFE) are emerging extraction technologies that are sustainable and environmentally friendly. The PLE method uses solvent extraction at high pressures and moderate temperatures, below the critical point of the solvent, so that the solvent remains in a liquid state throughout the extraction process [3]. Meanwhile, SFE begins with a fluid that is propelled at a pressure and temperature above the critical point, which becomes a supercritical fluid. Under these conditions, the properties of the fluid are somewhere between the properties of a gas and those of a liquid. Although the density of a supercritical fluid is similar to that of a liquid and its viscosity is similar to that of a gas, its diffusion is between the two states. Therefore, the supercritical state of a fluid has been defined as a state in which the liquid and gas cannot be distinguished one from another or as a state in which the liquid is compressible (i.e., behaves as a gas) [4,5].

Different plant matrices, spices and agro-industrial waste have been used as natural sources to extract pressurized and supercritical fluids. Legumes, vegetables [5], spices [6], aromatic plants, fruit, microalgae [7,8], berries seeds [9], and agro-industrial sub-products [10] have all been used to obtain natural antioxidant compounds.

Several studies have compared the antioxidant activity of plant extracts obtained by SFE and PLE. For example, 16 anthocyanins from extracts that contained phenolic compounds, antioxidants, and anthocyanins from blueberry processing waste were recovered and identified [9]. Such results offer promising prospects for the recovery of high-value components from plant residues using these technologies. This is a new possibility for the valorization and production of new healthy food-grade components.

Therefore, the objective of the present review was to analyze the design and development of the PLE and SFE technologies together with other emerging technologies from the point of view of their application in extracting bioactive components derived from different plant matrices and agro-industrial and marine life waste. These components can be valorized industrially by developing new ingredients for the formulation of healthy foods, nutraceuticals, and/or other products that promote consumer well-being.

## 2. Historical Background

**Figure 1:**
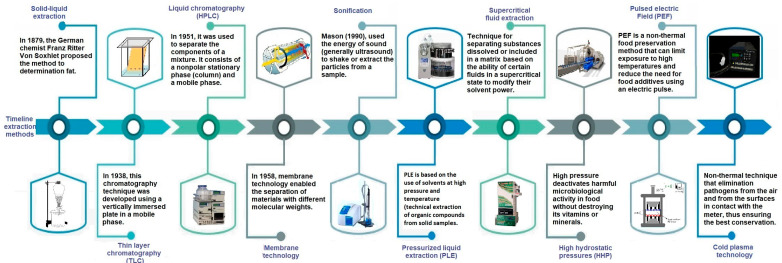
Development of extraction methods over time. (Schematic of High Performance Liquid Chromatograph (HPLC), model JASCO; Sonification SFX250/Branson Ultrasonics 101063966R; Pressurized Liquid Extraction (PLE), model ASE 350, Thermo Scientific™; Supercritical Fluid Extraction (SFE), model Spe-ed SFE-2, 7071, Appl Separation™; High Hydrostatic Pressures (HHP), model Hiperbaric 55 and Cold Plasma Technology, model PLasmaTact 15).

The PLE method can be considered a type of liquid-solid extraction, which was developed from Soxhlet extraction, which was discovered by the German inventor Franz Ritter von Soxhlet in 1879. This method was a breakthrough for obtaining solutes from materials that were difficult to recover from solid samples. For this reason, the Soxhlet method has become a reference standard in analytical extraction for more than a century. New extraction techniques were subsequently developed, such as SFE, PLE, and other techniques, which proved to be cleaner and/or more efficient as compared with Soxhlet extraction (Figure 1).

In principle, SFE is very similar to PLE. The first SFE patent applied to the food industry was developed in the United States in 1970, obtaining fluid extracts of hops with supercritical carbon dioxides (SC-CO_2_) and also caffeine-free coffee extract [11,12]. In 1994, a PLE technology known as subcritical water extraction (SWE) or pressurized hot water extraction was developed to extract organic contaminants from solid environmental samples [13]. Dionex introduced the first extraction equipment called accelerated solvent extraction (ASE) technology in 1995 [14]. In 1997, PLE was applied to recover taxanes from the Japanese yew (Taxus cuspidate) with methanol [15].

The first phenolic extract by PLE was obtained in 2001 from apple pulp and peel using methanol [16]. In 2009, it was developed a multipurpose type of extraction that combined SC-CO_2_ and SWE to recover oil from cashew shells [17]. As of 2004, PLE was combined with other techniques such as ultrasound, to obtain an isoflavone extract from various soybean by-products, producing higher yields than the Soxhlet method [18]. There was a significant trend to use PLE and SFE from 2011 to 2021.

## 3. Pressurized Liquid Extraction (PLE)

### 3.1. Principles of Pressurized Liquid Extraction (PLE)

The PLE method is a rapid extraction technology because of the direct interaction between the liquid solvent and the particles of the plant matrix under high pressure and subcritical temperature conditions to extract efficiently the compounds of interest. The efficiency of the extraction process mainly depends on the three interrelated aspects of the matrix, mass transfer, and solubility. Regarding the matrix, its nature and the compound of interest to be extracted and its location within the matrix have an influence [3]. Solubility properties and mass transfer between the plant matrix and extraction solvent greatly improve with increased temperature, which results in better extraction kinetics. Solvent viscosity decreases, which facilitates the hydration of the plant matrix and increases the solubility of the bioactive compounds. High temperature also causes the breakdown of bonds or bonding forces in the matrix (dipoles, van der Waals, and hydrogen bridges), facilitates the release of compounds, and produces high extraction yields [19].

### 3.2. Mechanism and Components of Pressurized Liquid Extraction (PLE)

**Figure 2:**
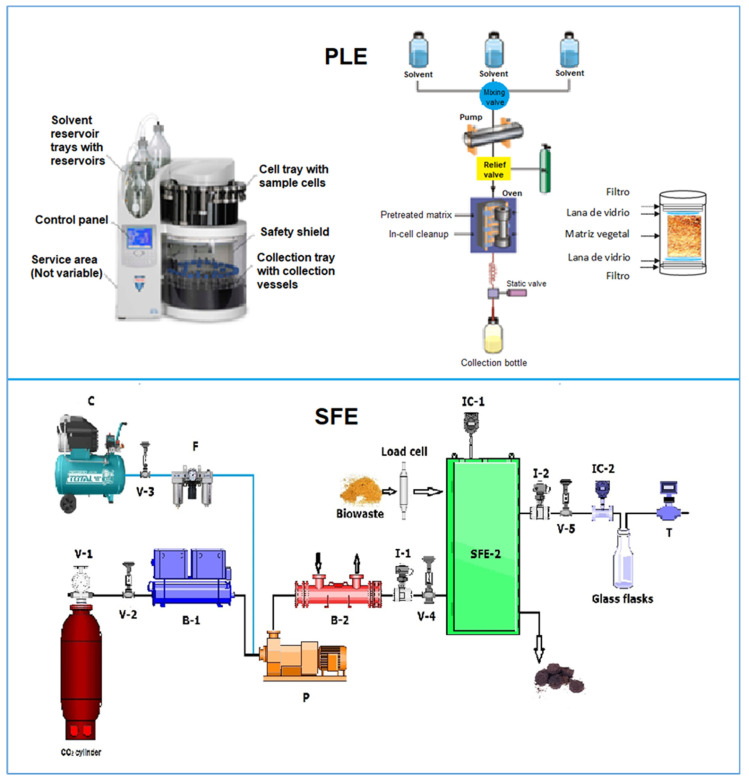
Schematic of pressurized liquid extraction (PLE, Dionex ASE 350, Thermo Scientific™) and supercritical fluid extraction (SFE). V-1, V-2, V-3, and V-4: Control valves; V-5: micrometric valve; C: compressor; F: compressed air filter; B1: water chiller; P: pump; B2: heating block; I-1 and I-2: pressure and temperature indicators, respectively; IC-1 and IC-2: extraction column temperature and micrometric valve temperature, respectively; EC: extraction column; T: flow meter.

The PLE process consists of circulating the solvent through the extraction cell or column with a high-performance liquid chromatography (HPLC) pump where the plant matrix is located to remove the bioactive compounds of interest (Figure 2). The previously prepared and conditioned sample in the extraction column is subjected to the selected temperature with an electrothermal liner and compressed to the specified pressure. Pressure and temperature are maintained constant to begin static extraction to stabilize the system and enable diffusion of the solvent through the plant matrix. Finally, the required pressure and solvent flow rate are maintained, and dynamic extraction begins. Extractions can be performed in more than one cycle; at the end of the process, the extraction column that contains the sample is removed and replaced by one that only contains an inert element. The system is then washed by pumping out the solvent and passing nitrogen or carbon dioxide through it [19].

### 3.3. Factors That Affect Pressurized Liquid Extraction (PLE)

#### 3.3.1. Temperature

Temperature is the main factor because it modifies the physical and chemical properties of solvents and influences extraction efficiency. Temperature decreases the dielectric constant of the solvent and changes its polarizability [3,20]. It also decreases solvent viscosity and density, increases diffusion and penetration into the matrix, and generates a higher mass transfer rate [3,19].

The use of high temperatures causes the breakdown of the structure of the vegetable matrix, and the reduction of the surface tension that occurs between the sample, the solvent, and the compounds during the extraction process. This is desirable because certain cavities are formed in the solvent, where the compounds of interest are transported through [21]. Likewise, the use of high temperatures can cause degradation of thermolabile compounds [3]. High temperatures (>150 °C) can generate the formation of toxic compounds such as hydroxymethylfurfural (HMF) [22]. Therefore, it is important to be able to know the nature of the compound to be extracted and to optimize the temperature to obtain high extraction yields, avoiding undesirable compounds.

#### 3.3.2. Pressure

A great advantage of PLE is that by using this high pressure technology, the solvents remain in a liquid state, even when subjected to temperatures above their boiling points. [23]. The highest pressures produce higher extraction yields because they help to hydrate the plant matrix by keeping the solvent in a liquid state. However, their effect is less than that of temperature because air bubbles can form at higher pressures, thus reducing the solubility of the compounds of interest [19].

#### 3.3.3. Solvent

To select the appropriate solvent, there must be a chemical affinity between the extraction solvent and the compound of interest to be extracted so that high diffusion and mass transfer occur, which results in high extraction yields. In addition, the extraction solvent must be non-toxic or with low toxicity (generally recognized as safe, GRAS), accessible, inexpensive, and easy to dispose of. The water-ethanol mixture is the most environmentally friendly solvent [19].

#### 3.3.4. Plant Matrix

The plant matrix usually undergoes certain treatments before extraction, such as traditional drying, freeze-drying, grinding and sieving. The type and conditions of drying directly affect the extraction performance of the compounds. The particle size of the sample favors mass transfer. A larger contact surface of the particles with the extraction solvent will improve the adsorption and separation of the compounds of interest. However, some studies recommend not reducing the size of the particles too much, to avoid the obstruction of the diffusion of the solvent due to the compaction of the sample [3,19].

It is also important to know the nature of the target compound in the plant matrix (state and moisture content). Several studies have reported that samples with a high moisture content can improve extraction efficiency compared to using dry samples. This may be due to the structural collapse of the cells in the drying process, which hinders the release of the compounds of interest. However, other studies have shown that the presence of water can compete with the extraction solvent, thus reducing the recovery of bioactive compounds in the plant matrix [19].

#### 3.3.5. Extraction Time

Extraction time is the time that the solvent is in contact with the substrate at a given temperature, pressure, and flow rate. The most specific parameter to understand the extraction time required for the complete extraction of bioactive compounds from plant matrices is the static or dynamic extraction method. The factors that affect it are the matrix structure, type of compound of interest, and temperature or pressure being used [3]. Another factor is the solvent flow rate, which is important when operating in dynamic extraction mode [13].

#### 3.3.6. Energy and Environmental Impact

The extraction process involves operating with high process pressures and temperatures, which means that energy expenditure is high. However, it is lower compared with other extraction methods such as SFE or the conventional Soxhlet. Comparative studies relating the ecological footprint of PLE and Soxhlet extraction of rosemary showed that the energy consumption (kWh) of PLE was lower than for Soxhlet, although extraction temperature for PLE was higher (183 °C vs. 78 °C for Soxhlet) [24].

#### 3.3.7. Chemical and Sensory

According to some reported studies, the chemical effects related to the type of solvent applied in the pressurized liquid extraction such as ethanol, and others can produce some undesirable compounds such as hydroxymethylfurfural (HMF), a non-enzymatic brown indicator. Similarly, HMF has been linked to the induction of precursors of colon cancer in mice, however, it did not generate toxicity in laboratory tests. The ethanol concentration had a significant impact on the overall performance of the PLE process. The lower the ethanol content of PLE extracts, the greater the recovery of phenolic acids and flavanols. Therefore, recovery is recommended at ethanol concentrations of 15%, 32.5% and 50% for phenolic acids, stilbenes and flavonols, respectively. At 150 °C and 32.5% ethanol, the extracts had the highest content of total polyphenols and antioxidant capacity [22,25].

## 4. Supercritical Fluid Extraction (SFE)

### 4.1. Principles of Supercritical Fluid Extraction (SFE)

The SFE method is a separation technology that uses supercritical fluids as a solvent. Each fluid is characterized by a critical point that is defined in terms of critical temperature and pressure. Supercritical Fluids above the point of critical temperature cannot liquefy regardless of the applied pressure, but they can reach a density close to the liquid state [26]. Carbon dioxide (CO_2_) is an ideal solvent for product extraction because it is non-toxic, non-explosive, readily available, and easy to eliminate from the extracted product. After all, it is gaseous at room temperature and normal pressure, which simplifies the recovery of the pure, solvent-free analyte [27].

### 4.2. Mechanism and Components of Supercritical Fluid Extraction (SFE)

Figure 2 shows the components and accessories of the SFE extractor. Depending on specific requirements, it is possible to differentiate between analytical tools and preparative systems (laboratory or industrial scale). Analytical systems are used to prepare samples; for example, chromatography obtains mg/g of extracts. Preparative systems extract grams of compounds from solid or liquid samples when operating at an experimental scale or kilograms at an industrial scale.

### 4.3. Factors That Affect Supercritical Fluid Extraction (SFE)

#### 4.3.1. Temperature and Pressure

Temperature and pressure can significantly influence extraction yield. Increasing temperature decreases the solvent density, thus reducing the solubility of the component to be extracted. It has generally been observed that increased pressure results in higher extraction yield because of the greater solubility of the components in the matrix. However, high pressure is not always recommended due to possible interactions between CO_2_ and the solute. In addition, the saturation pressure of the solute in SFE increases when temperature increases, and this improves solubility. Oil extraction significantly increases with increasing pressure at a given temperature. The authors state that an increase in pressure results in higher extract yields. Similarly, SFE density increases with SC-CO_2_ as well as its solubility. The extraction yield is significantly improved with an increase in pressure due to the increased solubility of components in the matrix [28,29].

Temperature seems to promote the rapid release of lipophilic extracts from the plant matrix, as can be seen after only 10 min for seed samples with the application of SC-CO_2_ [28]. The extraction pressure is one of the factors that influences the extraction efficiency. It was observed that oil production increases significantly with the increase of pressure at a given temperature. If the given temperature is higher than a certain value (around 45 °C), as the pressure increases, the oil production increases at lower pressure levels. Once the pressure reaches high levels, the production of extracts decreases slightly) [29].

It is more difficult to predict the effect of temperature on extraction compared to pressure due to its opposite effect on oil yield. First, an increase in temperature reduces the density of carbon dioxide, resulting in a decrease in the solvent’s capacity to dissolve the solute. Secondly, an increase in temperature increases the vapor pressure of the solutes, leading to an increase in the solubility of oils in SFE with carbon dioxide. Therefore, it is likely that solubility will decrease, remain constant, or increase as temperature increases at constant pressure, depending on whether the solvent density or the vapor pressure of the solute predominates [30].

#### 4.3.2. Particle Size and Plant Matrix

Particle size has a decisive influence on the extraction efficiency. The smaller the particles, the higher the extraction rate. The study of the extraction kinetics allows explaining the positive effect of particle size reduction on the internal resistance to mass transfer in the solid matrix. The extraction rate increased due to the shortening of the diffusion path. The crushing of the materials produces smaller particles leading to an increase in specific surface area as well as the breakdown of cell walls and other internal barriers preventing mass transfer. This effect makes lipophilic extracts more accessible for supercritical SC-CO_2_ extraction [31].

Intraparticle diffusion resistance is lower at smaller particle size. The intraparticle diffusion effect seems to become significant for larger particle sizes, resulting in a noticeable decrease in extraction. In this case, part of the extract is not extracted due to the long diffusion times of the solvent in the particles of the plant matrices. This implies a higher extraction rate by releasing and making the plant cell components more accessible during grinding. This effect may be greater with smaller particle sizes. Therefore, the extraction yield increases with decreasing particle size [31].

Both the particle size and the knowledge of the secretory structure of plant materials allow predicting the extraction behavior during the SFE process of essential oils. In the case of SFE of species with secretory ducts, particle size is not expected to affect the development of extraction yield, an important fact from the point of view of SFE for oils on an industrial scale [32,33].

#### 4.3.3. Extraction Time

The extraction time is a factor that affects the performance of treatment with supercritical fluids. In general, a longer extraction time allows for better dissolution of the analyte in the supercritical fluid and therefore a greater amount of extracted analyte. However, too long an extraction time can also lead to analyte degradation due to prolonged interaction with the supercritical fluid [30].

The extraction process consists of three stages related to the rapid extraction of free solute, surface and internal diffusion transition phase, and slow extraction based on internal diffusion. The time required for the first extraction stage depends on solute solubility in the extraction with SC-CO_2_ and on particle size [29].

The extraction time is also related to the kinetics of mass transfer between the sample and the supercritical fluid. As extraction progresses, the concentration of the analyte in the matrix decreases and therefore the mass transfer rate also decreases. This means that, after a certain point, the increase in extraction time will not be proportional to the improvement in extraction performance. In general, the optimal extraction time for an SFE analysis will depend on several factors, such as the nature of the analyte and sample matrix, temperature, pressure, and solvent-to-sample ratio. It is important to optimize extraction time for each analysis system for maximum extraction performance and the best quality of results [30].

#### 4.3.4. Co-Solvents

The affinity of CO_2_ for the extraction of non-polar components limits its use to obtaining polar or intermediate polarity compounds. Therefore, a small amount of a polar organic solvent (methanol, acetonitrile, water, etc.), called “co-solvents”, is added to the supercritical liquid to extract those compounds of polar nature. The nature of the solvent to be used as a supercritical fluid will depend on the nature of the solute to be extracted. [29].

It is important to note that the addition of a polar co-solvent to SC-CO_2_ may decrease the extraction yield. Most of the essential oil obtained is a nonpolar compound; therefore, due to the use of co-solvents, the decrease in its solubility in SC-CO_2_ causes the extraction yield to decrease [29]. By adding a co-solvent such as methanol or ethanol, it is possible to lower the temperature, lower the pressure, reduce the extraction of undesired components (such as free fatty acids, alcohols, and waxes) and increase the extraction efficiency in SFE [34].

The addition of large amounts of co-solvents can significantly change the critical parameters of the mixture. As a result, binary carbon dioxide-organic solvent mixtures are used in the supercritical state, where diffusion coefficients are lower than in the supercritical state [34].

## 5. Recovery of Bioactive Components by Pressurized Liquid Extraction (PLE) and Supercritical Fluid Extraction (SFE) from the Food Matrix and Agro-Industrial Waste

In recent years, several studies have been conducted by PLE and SFE to obtain by-products or agricultural products of bioactive compounds from various food matrices such as vegetables, fruit, herbs, spices, microalgae, algae, and cereals. It has been demonstrated that high extraction rates can be obtained by these two extraction methods. Table 1 represents most of the work performed by different researchers. Food matrices and waste treated by SFE and PLE with all the optimal extraction parameters are described. This reference is a great contribution to managing new experiments and comparing both technologies according to the extracted functional components.

## 6. Application of Bioactive Compounds as Ingredients in Health Food Formulation and Their Preventive Effect on Health

### 6.1. Bioactive Compounds and Health Effect

The bioactive compounds obtained by SFE and PLE have several characteristics that are suitable for use as an ingredient in a value-added functional food product. Firstly, these compounds have a protective effect (edible coating) and secondly, it is food additives (value-added products). Finally, it is a modifier of the final composition of the mixture.

Table 2 describes several compounds related to their source applied to food that greatly modifies the beneficial effects associated with health. Several bioactive compounds belong to the phenolic compounds. These compounds have demonstrated antioxidant, chelating, cardioprotective, and anticarcinogenic activity [84,85,86,87]. There is evidence of many matrices with flavonoids and anthocyanins, which have been shown to have an immunoregulatory and anti-inflammatory effect at the gastrointestinal level [88,89]. Rosmarinic acid has shown a protective effect against cardiovascular diseases and some types of cancer [90]. Finally, punicalagin, an ellagitannin, has been shown to have an effect in the control and prevention of cardiovascular diseases, diabetes, and prostate cancer [91].

In this aspect, SFE and PLE technologies can contribute to obtaining phenolic compounds from different food matrices and allow the reformulation of products with functional characteristics that satisfy consumer trends for this type of food. In fact, according to the portal Mordorintelligence.com, by 2025, the market for foods with antioxidant activity is expected to reach 1.8 billion dollars, with the highest growth in the Asia-Pacific region.

In the case of carotenoids, their intake has been associated with the prevention of certain types of diseases such as diabetes, cancer, and a decrease in obesity rates. Some studies have even demonstrated their prebiotic activity [125]. Curcumin, on the other hand, has been shown to have anti-inflammatory effects and to improve the quality of life and health status in cancer patients and has even been shown to improve the mental health of patients with dementia, anxiety, and depression [126].

Table 2 shows the great potential of extracting bioactive compounds from some food matrices and even from their residues and components that are not normally used in the industry. However, for many of these matrices and their bioactive compounds, their possible applications for the food industry have not yet been studied, as is the case for most of the bioactive extracts from algae, fruit peels, seeds, and bagasse. Although, their health effects have been studied in recent years and some applications have been reported in other sectors such as animal feed, pharmaceuticals, cosmetics, composting, fertilizers or biorefinery [127]. For example, in the case of algae, pigments and phenolic compounds with antioxidant, anticancer, antimicrobial, and antihypertensive activity have been identified [128,129].

In this context, there remains a large field to be further explored and studied, with the use of new coupled technologies such as PLE and SFE that allow the extraction of bioactive compounds and from these can develop various applications with functionality in the food industry.

### 6.2. Food Waste and Novel Bioactive Compounds

**Figure 3:**
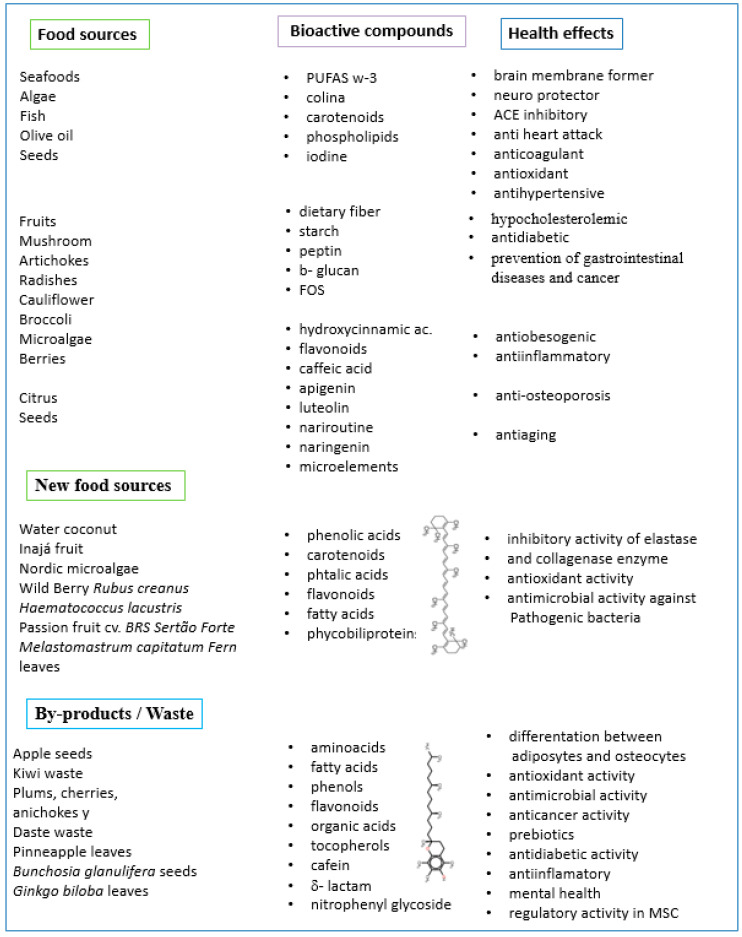
Bioactive compounds extracted from new food sources or residues of agrochains and their effect on health.

In recent years, research has given relevance to the use of waste generated in food production chains, as well as to the identification of new varieties of “local” foods and the use of parts that were normally discarded in the industry to identify their potential use. Many of these new foods and residues have a significant number of bioactive compounds with potential beneficial effects on human health. In this context, the technologies addressed in this paper (PLE and SFE) could contribute to the extraction of these bioactive compounds for use as raw materials or ingredients in new food or packaging formulations, as will be presented in this section (Figure 3).

Regarding new sources of bioactive compounds, there is a lot of interesting research. The analysts studied for example: coconut water from different Malaysian varieties as a new source of bioactive compounds with antioxidant and anti-aging activity. Among the bioactive compounds, they highlighted gallic acid, catechin, trans-zeatin, dihydrozeatin, kinetin, kinetin riboside and trans-zeatin riboside and showed their elastase and collagenase enzyme inhibitory activity [130].

The bioactive compounds of a traditional Brazilian inajá fruit (*Maximilia maripa*) were studied, which has been underutilized in the country. The authors identified several bioactive compounds such as gallic acid, 4-dihydroxybenzoic acid, ferulic acid, *p*-coumaric acid, and m-coumaric acid and demonstrated the antioxidant activity of the fruit extracts [131]. Also in Brazil, the bioactive compounds of a new variety of passion fruit cv. BRS Sertão Forte (*Passiflora cincinnata Mast*) were evaluated. In this fruit they evidenced isoquercetin, caftaric acid and rutin, which showed antioxidant activity [132].

On the other hand, the cytotoxic and anticarcinogenic potential of new bioactive compounds isolated from the leaves of *Melastomastrum capitatum Fern* were assessed. The isolated compounds corresponded to fatty acid and phthalic acid derivatives such as Di-butyl phthalate, Di-benzofuran,3-nitro, phthalic acid-di(2-propylpentyl) ester, 9-octadecenoic acid *E*-oleic acid, and 2,4,7-trioxabicydo (4.4.0)9-decene,8(4-(4-pentyl cyclohexyl)cyclohexyloxy)-3-phenyl. The latter has never previously been isolated from any plant [133].

León-Vaz et al. studied another new source of bioactive compounds in 2023. Discovering that Nordic microalgae are a potential source of carotenoids such as neoxanthin, violaxanthin, antheraxanthin, lutein, c-lutein, astaxanthin, β-carotene, chlorophyll b, chlorophyll a, polyphenols and compounds with antioxidant activity [134]. Also, were investigated wild Berry *Rubus coreanus* as a new source of bioactive compounds with antioxidant and antimicrobial activity against pathogenic bacteria such as *S. enteritidis*, *V. cholerae*, *E. coli, S. barbata, S. typhimurium, and S. aureus* [135].

In the case of the microalga *Haematococcus lacustris*, its potential antimicrobial use was studied, especially as an alternative to antibiotics and pathogenic bacteria that have generated great resistance. The authors also identified bioactive compounds such as carotenoids, fatty acids, and phycobiliproteins [116].

As for the incorporation of residues or parts not commonly used, the effect of apple seeds as a source of amino acids, fatty acids and polyphenolic compound was investigated [136]. Among the most predominant fatty acids in the seed, linoleic acid and oleic acid stand out. Phytochemicals such as α and β-tocopherols, chlorogenic acid, Procyanidin B1, catechin, epicatechin, gallic acid and caffeic acid have also been identified. As well as anti-diabetic, anti-obesity, antioxidant, and antimicrobial properties have been identified in the seeds.

Chamorro et al. conducted a review of bioactive compounds in the kiwifruit agro-chain to valorize the residues generated, especially from leaves, stems, skin, and flowers. They showed the potential use of the residues as they contain flavonols, phenolic acids, anthocyanins, tocopherols, and organic acids such as malic, citric, and ascorbic acids. They also identified potential beneficial effects on health, such as anticancer, antioxidant, antidiabetic, and prebiotic activity [137].

Through this study, a review of the by-products of plums, cherries, artichokes and dates was carried out. They evidenced the presence of compounds with biological activity such as ellagic acid, epicatechin, Naringenin-7-*O*-glucoside, Luteolin-7-*O*-rutinoside, Apigenin, Chlorogenic acid/5-*O*-caffeoylquinic acid and β-Carotene. The by-products analyzed corresponded to stems, seeds, skin, leaves and flowers and evidenced anticancer, antioxidant, antimicrobial, antidiabetic, anti-inflammatory and antihyperglycemic potential [138].

Recently, the bioactive compounds present in pineapple leaves, as well as their effects on health, were evaluated. They evidenced some organic acids such as threonic acid, quinic acid, malic acid hexoside, malic acid, citric acid, and pipecolic acid, flavonoids such as apigenin and quercetin-3-*O*-rutinoside (rutin) [139].

They were also analyzed the seeds of *Bunchosia glandulifera*, evidencing the presence of phenolic compounds, caffeine, and δ-lactam, which they associated with the beneficial effects on mental health described by populations consuming this product [140]. According to Lee et al., identified a new nitrophenyl glycoside, ginkgonitroside, which they isolated from Ginkgo biloba leaves. This compound demonstrated regulatory activity on MSC differentiation between adipocytes and osteocytes [141].

## 7. Advantages and Disadvantages of Pressurized Liquid Extraction (PLE) and Supercritical Fluid Extraction (SFE)

### 7.1. Advantages and Disadvantages of PLE

The advantages of this method are evidenced by taking a solvent to the necessary pressure and temperature to act on plant molecules, achieving their extraction by mass transfer and solubility [142]. The use of pressurized liquid extraction employs environmentally friendly and GRAS-type solvents such as water and alcohol. It also uses a smaller volume of extraction solvent compared to conventional techniques, which in turn use toxic solvents [143].

This extraction method is performed in easy-to-use, practical, and simple equipment. Many of them have semi-automatic designs that can be coupled to analytical measurement and separation instruments, such as liquid phase chromatography (HPLC) and gas chromatography (GC) [19]. It is also worth noting that this is an emerging technology that can be coupled with other technologies such as supercritical fluid extraction (SFE) and ultrasound technology, which benefits from higher extraction efficiency [141,143].

On the other hand, the main disadvantage is its high energy consumption to reach the high system pressures, which increases production costs. It is not very selective due to the use of organic solvents, which only carry polar compounds, although it could be enriched with the use of modifiers to alter the polarity of the latter. It can be used to extract thermolabile analytes. As for the operational aspects, leaks in the system and obstructions in the piping can occur.

### 7.2. Advantages and Disadvantages of SFE

The advantages consist in the fact that the properties of supercritical fluids can be adjusted by varying the pressure and temperature [42]. Supercritical fluids have the advantage of being recyclable by reversing the change in temperature and pressure to extract a given substance from a food matrix [144]. Extraction with supercritical fluids allows obtaining solvent-free extracts and the extraction is faster than with the use of conventional organic solvents, all without leaving toxic residues as a key technology in “green chemistry” [74]. Using CO_2_, the treatment is performed at a moderate temperature (30–50 °C) and it is possible to achieve high selectivity of valuable microcomponents in natural products. CO_2_ selectivity is also suitable for the extraction of essential oils, pigments, antioxidant carotenoids, antimicrobials, and related substances [145]. These components were obtained by various studies cited in Table 1.

On the other hand, the main disadvantage is that cuticular waxes and high molecular weight compounds are extracted together with the essential oil, leading to additional effort to separate them [29]. Due to the nature of the process, high pressures are required to carry out the extraction. In addition, it requires high cost due to the initial investment for equipment and maintenance, solvent compression requires elaborate recirculation measures to reduce energy costs, it dissolves few polar compounds, and the use of modifiers can alter the polarity of CO_2_, requiring a subsequent separation process in the extract obtained [1,2,3,4].

### 7.3. Comparison between SFE and PLE

The use of both emerging technologies is applicable in obtaining useful compounds as functional ingredients. There are a few differences to highlight in terms of cost, operational process, and selectivity. The comparison between SFE and PLE in economic terms seems to be the key factor for the choice of the most convenient method. The investment and operational costs of a PLE plant are lower than those of an SFE since in the first case the pressurization section of SC-CO_2_ is not necessary, and the extraction is faster. Therefore, since pressurized solvent extraction takes less time, is cheaper to use, and has more advantages over SFE (green solvents and moderate temperatures), it should be considered a promising alternative to extract phenolic compounds or other hydrophilic compounds. Further studies could be interesting to optimize this method and possibly obtain a higher phenolic yield [6].

However, the criteria may vary greatly from one author to another. The compounds obtained by free ellagic acid (FEA) from thyme contained 70–80 mg/g of thymol, whereas very low amounts of thymol were obtained in the PLE samples [146]. Besides considering that both methods are very efficient in obtaining oil rich in alpha-linolenic acid (ALA) of low-quality chia seed (impurities, yellowish color, high humidity and unknown origin) [147]. PL proved to be more efficient than SFE removing a wide variety of compounds with different polarities, with phenols being the most abundant, while SFE resulted in a higher number of fatty acids and derivatives. One option is the combination of both methods, which allows obtaining a more complemented final product [42].

## 8. Future Expectations on the Use of Pressurized Liquid Extraction (PLE) and Supercritical Fluid Extraction (SFE) Technology

### 8.1. Sequential Biorefining of Bioactive Compounds and Components by SFE-PLE

**Figure 4:**
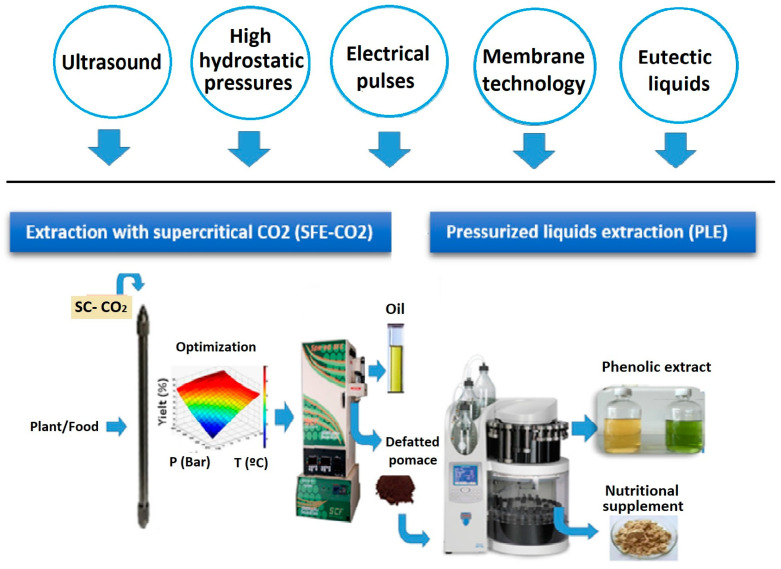
Future possibilities of interaction between SFE and PLE technologies for the biorefinery of food waste to obtain bioactive components (Modified scheme based on previous studies) [37]. Scheme based on PLE and SFE equipment (Dionex ASE 350, Thermo Scientific™; Spe-ed SFE-2, 7071, Appl. Separation™, respectively).

Figure 4 illustrates a schematic of the biorefinery of bioactive components from waste by alternating the combination of the SFE and PLE techniques. The combination of both techniques makes it feasible to completely use and separate all bioactive compounds from waste [148]. For example, obtained extracts from cherry stems by SFE-PLE; they identified 42 compounds by HPLC-ESI-QTOF-MS, of which 22 were unknown and feasible for incorporating in food formulations and nutraceuticals described several technologies for the enhanced extraction of bioactive carbohydrates that comply with the principles of green chemistry and combine many emerging technologies [37,149]. A good example is the possible combinations of ultrasound with a wide variety of emerging eco-friendly technologies at small and medium scales [150]. Furthermore, technological developments in equipment designs, especially at an industrial level, continue to be necessary and can significantly contribute to broadening the application of these methodologies in various fields. Finally, a better understanding of the mechanisms underlying recently developed extraction methods is crucial for promoting their use as cost-effective and environmentally friendly techniques to obtain compounds that are rich in bioactive extracts. To provide an increased performance of these technologies, a variety of new combinations of green solvents (DES, NADES, etc.) have recently emerged to achieve a better and more selective extraction, while complying with the sustainability principles (recycling, low toxicity, low energy consumption, etc.) of green chemistry.

### 8.2. Microencapsulation and Nanoencapsulation by Pressurized Liquid Extraction (PLE) and Supercritical Fluid Extraction (SFE)

**Figure 5:**
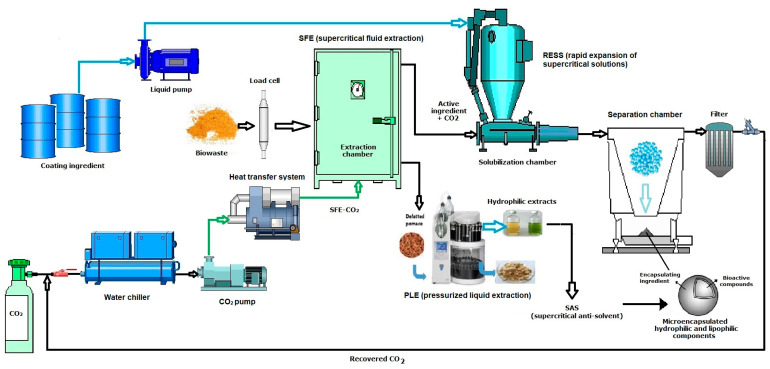
Combination of supercritical fluid extraction (SFE) and pressurized liquid extraction (PLE) technology to obtain microencapsulates by SAS (supercritical antisolvent) and RESS (rapid expansion of supercritical solutions) from agro-industrial biowaste. Scheme based on PLE equipment (Dionex ASE 350, Thermo Scientific™).

The use of SFE as a microencapsulation tool for RESS (rapid expansion of supercritical solutions) is a great technological contribution; active and coating ingredients are dissolved in a supercritical fluid that acts as a solvent. The supercritical fluid solution that contains the solutes is maintained at high pressure before expanding through a capillary device or orifice nozzle. At this point, supersaturation occurs and leads to the solvation of the coating material that is deposited around the active ingredient to form microcapsules [151]. In addition to obtaining bioactive compounds, the preservation of lipid extracts in microcapsules useful when applied to any functional food is achieved (Figure 5). There are also other techniques such as SAS (supercritical antisolvent) or SCMM (supercritical melt micronization) [152], microencapsulation by coprecipitation (RESS, SAS, supercritical spray drying) [153] and active coating ingredient (spray coating, fluidized bed coating) with SC-CO_2_ [154]. The supercritical fluid can be used in many ways to produce microencapsulated and nano-encapsulated products depending on the properties of the active ingredient, coating material, and appropriate solvent to be used [151].

Although SFE technology has a direct impact on microencapsulation and nanoencapsulation mechanisms, it should be noted that PLE greatly benefits by obtaining bioactive compounds for spray-drying treatment, thus providing good encapsulation (Figure 5). This well-established method for particle formation, including recent advances in encapsulation, has shown excellent results for compound stability in the presence of oxygen, light, or high temperatures, which extends its use as a food additive or ingredient [155,156].

## 9. Conclusions

Supercritical fluid extraction (SFE) and pressurized liquid extraction (PLE) are extraction techniques that are considered environmentally friendly. They use solvents such as water, ethanol, or CO_2_ to produce harmless extracts from plant substrates or industrial biowaste. When these technologies are combined, they greatly improve the mass transfer efficiency of the extraction process; this favors solvent penetration into the matrix, and extraction rate, and shortens extraction time compared with traditional extraction methods. The potential of these methodologies has a wide range of applications that encompass the food, pharmaceutical, and cosmetic industries. In addition, these food-grade extracts are ingredients that provide a protective, modifying, and nutritional additive effect. Beneficial effects include cholesterol reduction, prevention of gastrointestinal problems, and anticancer, antimicrobial, and antioxidant properties.

The scale-up of these technologies and their combination with other emerging processes ensure a promising tool in the short term for processing biowaste and generating various liquid extracts (lipophilic and hydrophilic), microencapsulated or nano-encapsulated powders, safe products, and other applications. These provide sustainable production of the food industry in line with the trends and needs to implement the global circular economy.

## Figures and Tables

**Table 1:** Optimal extraction parameters by pressurized liquid extraction (PLE) and supercritical fluid extraction (SFE) of food matrices and agro-industrial waste.

Food Matrices and Residues	Group and Bioactive Compound of Interest (PLE)	Optimal Extraction Parameters (PLE)	Group and Bioactive Compound of Interest (SFE)	Optimal Extraction Parameters (SFE)	References
Vegetables and fruit					
Asparagus (*Asparagus officinalis* L.)	Phenolic compounds (APM) Phenolic acids (3-*O*-feruloylquinic acid)	S = Water/Ethanol (1:1) T = 65 °C and t = 30 min P = 100 MPa FR = 2 mL/min	Phenolic contents (BPM) Phenolic acids (3-*O*-feruloylquinic acid)	S = CO_2_ + cosolvent - (1:1) T = 65 °C and t = 60 min P = 15 MPa FR = 0.25 kg/h	[23]
Spinach (*Spinacia oleracea* L.)	Polyphenols Phenolic compounds	S = Water/Ethanol (1:1) T = 80 °C and t = 10 min P = 10.3 MPa	Carotenoids Phenolic compounds	S = CO_2_ T = 70 °C and t = 6 h P = 25 MPa FR = 60 g/min	[35]
Parsley and seeds (*Petroselinum crispum*)	Phenolic compounds (Apin and Malonyl-apin)	S = 50% Ethanol T = 70 °C (Malonyl-apin), 160 °C (Apin) and t = 5 min P = 6.9 MPa PS = <0.425 mm S/RM = 40	Phenylpropanoids (Apiol) (82.1%) Myristicin (11.4%) Essential fatty acid	S = CO_2_ T = 40 °C P = 90–300 bar	[36]
Blackberries and leaves (*Morus nigra* L.)	Anthocyanins	S = Water T = 60 °C and t = 60 min P = 15 MPa FR = 2 mL/min	Phenolic acids Flavonoids	S = CO_2_ + cosolvent T = 40 °C and t = 120 min P = 150–300 bar FR = 22 g/min	[37]
Goji berry (*Lycium barbarum* L.)	Flavonols Phenolic acids	S = 86% Ethanol T = 180 °C and t = 5–20 min P = 10 MPa	---	---	[38]
Juçara and its residues (*Euterpe edulis* Mart.)	Anthocyanins	S = Water/Ethanol (50:50) T = 30 °C and t = 5–11 min P = 2 MPa S/RM = 8.5	Anthocyanins Phenolic compounds	S = CO_2_ + cosolvent T = 60 °C P = 20 MPa FR = 2.08 × 10^−4^ kg/s S/RM = 90	[39]
Herbs or spices					
Rosemary (*Rosmarinus officinalis* L.)	Phenolic diterpenes (carnosol, scutellarein) Flavonoids (genkwanin)	S = Water T = 100 °C and t = 15 min P = 6 MPa FR = 1 mL/min	Diterpenes (PDA) Carnosic acid Carnosol. a-pinene, 1,8-cineole, verbenone, camphor, borneol, and others	S = CO_2_ + cosolvent T = 60 °C and t = 45 min P = 250 atm FR = 1 mL/min	[40,41]
Felty germander (*Teucrium* *montanum* L.)	Flavanone (naringin) Flavonoids Flavan-3-ols (catechins and epicatechins) Flavonol (routine) Gallic acid	S = Water T = 160 °C and t = 30 min P = 1 MPa S/RM = 10	---	---	[42]
Gotu kola (*Centella asiatica*)	Asiatic acid and asiaticoside	S = Water T = 250 °C and t = 300 min P = 40 MPa	---	---	[43]
Lemon balm (*Melissa officinalis*)	Rosmarinic acid and its derivatives	S = Water T = 150 °C and t = 20 min P = 6.9 MPa	Rosmarinic acid and its derivatives	S = CO_2_ + cosolvent T = 60 °C and t = 60 min P = 40 MPa	[44,45]
Cinnamon (*Cinnamomum* *zeylanicum*)	Phenolic acids (caffeic, ferulic, p-coumaric, protocatechuic, and vanillic)	S = Water T = 200 °C and t = 60 min P = 6 MPa FR = 3 mL/min	(E)-cinnamaldehyde, (E)-β-caryophyllene, α-terpineol, and eugenol.	S = CO_2_ T = 50 °C and t = 60 min P = 90 bar FR = 6 kg/h	[46]
Horsetail (*Equisetum* *arvense L.*)	Phenolic acids (chlorogenic, caffeic, ferulic) Flavonoids (isoquercitrin, 5-glucoside luteolin)	S = 80% Methanol T = 80 °C and t = 30 min (3 cycles/10 min) P = 6 MPa	---	---	[47]
Chanca piedra (*Phyllanthus)* *amarus*)	Gallic acid Hydrolysable tannins Flavonoids Lignans	S = Water T = 192.4 °C and t = 15 min P = 11 ± 0.7 MPa S/RM = 24	Ellagitannins (hydrolysable tannins) Flavonoids (condensed tannins)	S = CO_2_ + cosolvent T = 60 °C and t = 4 h P = 200 bar FR = 1.5 mL/min S/RM = 72	[48]
Turmeric (*Curcuma* *longa* L.)	Curcumin	S = Water T = 140 °C and t = 14 min P = 1 MPa PS = 0.71 mm	Turmerones (turmerone, ar-turmerone, curlone)	S = CO_2_ T = 40 °C P = 9–66 MPa FR = 1.8 g/min	[49,50]
Microalgae or macroalgae					
Brown macroalga: Japanese wireweed (*Sargassum muticum*)	Phenolic compounds (phlorotannins)	S = Ethanol/Water (75:25) T = 120 °C and t = 20 min P = 10.3 MPa	Phenolic compounds (mannitol)	S = CO_2_ + cosolvent T = 60 °C and t = 90 min P = 15.2 MPa	[51]
Green microalga (*Haematococcus pluvialis*)	Short-chain fatty acids (vitamin E) Simple phenols (gallic acid)	S = Water T = 200 °C and t = 20 min P = 10.3 MPa	Astaxanthin Omega-6 and omega-3 Unsaturated fatty acids (UFA)	S = CO_2_ T = 50 °C and t = 95 min P = 50 MPa FR = 4 L/min	[52]
Spirulina microalga: (*Spirulina platensis*)	Carotenoids (zeaxanthin and β-carotene) Phenolic compounds Chlorophyll	S = Ethanol T = 115 °C and t = 15 min P = 6.9 MPa	Carotenoids Gamma-linolenic acid	S = CO_2_ T = 40–50 °C and t = 4 h P = 25–70 MPa FR = 10 kg/h	[10,53]
Royal Kombu (*Laminaria* *japonica Areschoug*)	Carotenoids Chlorophyll	S = Ethanol with 4.73% R134a T = 51 °C and t = 15–50 min P = 17 MPa FR = 10 g/min	---	---	[54]
Green microalga (*Chlorella vulgaris*)	β-Carotene Chlorophyll a and b	S = 90% Ethanol T = 116.8 °C and t = 25.1 min T = 160–173 °C and t = 14.7 min P = 10.3 MPa	Carotenoids	S = CO_2_ + cosolvent T = 40 °C and t = 30 min P = 300 bar FR = 0.34 L/min	[55]
Microalgae *Chlorella* spp.	Phenolic acids (p-coumaric, ferulic, and caffeic)	S = Water T = 163 °C and t = 5 min Microalga concentration = 20%	Lipids Palmitic acid	S = CO_2_ T = 60 °C P = 306 bar	[56]
Grains					
Rice (*Oryza sativa*)	Protocatechuic, vanillic, ellagic, and guaiacol acids	S = 60% Acetic acid and ethyl acetate in methanol T = 190 °C and t = 10 min P = 20 MPa	Volatile aromatic components	S = CO_2_ T = 50 °C and t = 120 min P = 120 bar FR = 0.1 to 0.4 L/min	[57]
Flaxseed (*Linum* *Usitatissimum* L.)	Phenolic compounds Lignans (SDG) Flavonoids	S = Water T = 180 °C and t = 15 min P = 10.3 MPa	Fatty acids (linolenic and oleic acid)	S = CO_2_ + cosolvent T = 323 K and t = 5 h P = 25 MPa PS = 0.42 × 10^−3^ FR = 1.7 × 10^−5^ kg/s	[58]
Sesame (*Sesamum indicum* L.) Husks	Phenolic compounds Lignans	S = 63.5% Ethanol T = 220 °C and t = 50 min P = 8 MPa FR = 5 g/min	Essential oils (Oleic, linoleic, stearic, and palmitic acids)	S = CO_2_ T = 35 °C and t = 210 min P = 20 MPa FR = 2.5 g/min	[59]
Onion peel (*Allium cepa*)	Flavonols (quercetin)	S = Water T = 165 °C and t = 15 min P = 9–13.1 MPa	Polyphenols (gallic acid) Flavonoids		[60]
Orange peel (*Citrus x sinensis*)	Flavonoids Glycosylated (hesperidin)	S = 75% Ethanol T = 65 °C and t = 40 min P = 10 MPa FR = 2.37 g/min S/RM = 47	β-myrcene, 1,8-cineole, D limonene, dihydrocarveol, and caryophyllene.	S = CO_2_ + cosolvents T = 60–75 °C and t = 90 min P = 90 MPa FR = 34.5 cm/s	[61,62]
Pomegranate peel (*Punica granatum* L.)	Punicalagin	S = Ethanol T = 200 °C and t = 20 min P = 10.3 MPa	Phenolic compounds	S = CO_2_ T = 46.5 °C and t = 2.5 h P = 291 bar FR = 2 L/min	[63,64]
Aguaje peel (*Mauritia flexuosa* L.)	Carotenoids Polyphenols	S = 92.43% Ethanol T = 37.7 °C and t = 90 min P = 10 MPa FR = 3 mL/min	Essential oil Carotenoids	S = CO_2_ T = 45 °C P = 300 bar	[65]
Avocado peel (*Persea americana* Mill.)	Procyanidins Flavonols Hydroxybenzoic and hydroxycinnamic acids	S = Water/Ethanol (1:1) T = 200 °C and t = 20 min P = 11 MPa	Thirteen compounds identified in essential oil	S = CO_2_ T = 50 °C and t = 40 min P = 250 bar FR = 10 mL/min	[66]
Tangerine peel (*Citrus unshiu* Marcow)	Flavonoids (hesperidin, naringin, naringenin)	S = Water T = 130 °C and t = 15 min P = 3 MPa S/RM = 34	Flavonoids (naringin, hesperidin, sinensetin, nobiletin, and tangeretin)	S = CO_2_ + cosolvents T = 80 °C and t = 210 min P = 22 MPa FR = 9 g/min	[67]
Genipap peel (*Genipa americana* L.)	Iridoids (genipin and geniposide)	S = Ethanol T = 50 °C and t = 5 min P = 0.2 MPa S/RM = 5	Fatty acids (palmitic, stearic, linoleic, and linolenic acids)	S = CO_2_ + cosolvents T = 333 K P = 30 MPa FR = 2.5 ± 0.5 g/min S/RM = 20 g	[68]
Mango peel (*Mangifera indica* L.)	Phenolic compounds	S = Water T = 180 °C and t = 90 min P = 10 MPa FR = 6.67 g/min S/F = 40	Bioactive compounds (gallotannins, flavonoids, xanthones, gallic acid, etc.)	S = CO_2_ + cosolvents T = 50 °C and t = 20 min P = 20 MPa FR = 2 L/h	[69]
Passion fruit peel (*Passiflora edulis*)	Phenolic compounds (Isoorientin, vicenin, vitexin, orientin, and isovitexin)	S = 70% Ethanol T = 60 °C and t = 30 min P = 10 ± 0.5 MPa FR = 2.7 mL/min	Fatty acids Carotenoids Tocols (tocopherols and tocotrienols)	S = CO_2_ + cosolvents T = 40 °C and t = 30 min P = 35 MPa FR = 0.63 kg/h S/RM = 46	[1]
Seeds					
Avocado seed (*Persea americana*)	Phenolic compounds Condensed tannins Phenolic acids Flavonoids	S = Water/Ethanol (1:1) T = 200 °C P = 11 MPa	Thirteen compounds identified in essential oil	S = CO_2_ T = 50 °C and t = 40 min P = 250 bar FR = 10 mL/min	[66,70]
Grape seed (*Vitis* *vinifera*)	Catechins Proanthocyanidins	S = Water T = 150 °C and t = 30 min P = 10.3 MPa	Essential oils (α-tocopherol)	S = CO_2_ + cosolvents T = 80 °C and t = 7 h P = 300 bar FR = 15 g/min PS = 300–425 μm	[71,72]
Papaya seed (*Carica papaya* L.)	Phenolic acids (Ferulic, mandelic, and vanillic)	S = Water T = 150 °C and t = 5 min P = 10 MPa FR = 4 mL/min	Phenolic compounds	S = CO_2_ + cosolvents T = 50 °C and t = 180 min P = 320 MPa FR = 0.50 ± 0.05 kg/h PS = 0.300 and 0.850 mm	[73,74]
Pomegranate seeds (*Punica granatum* L.)	Phenolic compounds (Caffeic acid derivatives and kaempferol 3-*O*-rutinoside)	S = Water T = 220 °C and t = 30 min P = 6 MPa S/RM = 40	Fatty acids (palmitic, stearic, oleic, linoleic, and punic acids)	S = CO_2_ T = 47 °C and t = 2 h P = 38 MPa FR = 21 L/h TP = 0.3mm	[75]
Genipap seeds (*Genipa americana* L.)	Iridoids (Genipin and geniposide)	S = Ethanol T = 50 °C and t = 5 min P = 1.2 MPa S/RM = 5	Fatty acids (palmitic, stearic, linoleic, and linolenic acids)	S = CO_2_ + cosolvents T = 333 K P = 30 MPa FR = 2.5 ± 0.5 g/min S/RM = 20 g	[68]
Bagasse					
Blackberry bagasse (*Rubus* spp.)	Phenolic compounds Monomeric anthocyanins	S = 50% Ethanol T = 100 °C and t = 30 min P = 7.5 MPa S/RM = 18 FR = 3.35 mL/min	Phenolic compounds Monomeric anthocyanins	S = CO_2_ + cosolvent T = 40–60 °C and t = 120 min P = 15 MPa S/RM = 400 FR = 2.77 × 10 ^−4^ kg/s PS = 0.34 mm	[76]
Grape pomace (*Vitis* *vinifera L. ‘Carménère’*)	Phenolic acids Flavanols Stilbenes	S = 15% Ethanol (acids), 32.5% (flavanols), and 50% (stilbenes) T = 150 °C and t = 30 min P = 10.3 MPa	Polyphenols and Vitamins (trans-resveratrol, β-sitosterol, α-tocopherol, and ascorbic acid)	S = CO_2_ + cosolvent T = 60 °C and t = 15 min P = 250 bar FR = 2 mL/min and 0.4 mL/min	[77]
Green kiwi bagasse (Actinidia deliciosa ‘Hayward’)	Phenolic compounds (catechin, chlorogenic acid, p-coumaric acid, protocatechuic acid, and caffeic acid)	S = Water T = 200 °C and t = 90 min P = 5 MPa		S = CO_2_ T = 80 °C and t = 5 min P = 300 atm FR = 3 mL/min	[78]
Red wine grape bagasse (*Vitis palmata* ‘Petit Verdot’)	Phenolic compounds	S = 50% Ethanol T = 120 °C and t = 90 min P = 9 MPa FR = 5 g/min	Phenolic compounds	S = CO_2_ + cosolvent T = 55 °C and t = 3 h P = 100 bar FR = 25 g/min	[79,80]
Blueberry bagasse (*Vaccinium myrtillus* L.)	Anthocyanins	S = Acidified water T = 40 °C and t = 15 min P = 20 MPa FR = 10 mL/min	Phenolic compounds Anthocyanins	S = CO_2_ T = 40 °C P = 25 MPa FR = 1.05 × 10^−4^ kg/s	[81]
Sugarcane bagasse (*Saccharum officinarum*)	Arabinoxylan Xylan	S = Water/0.1M NaOH T = 150 °C and t = 22 min P = 100 bar	Cane wax, oil, and resin (policosanol and octacosanol)	S = CO_2_ T = 323–333 °K and t = 0.5–2 h P = 20–35 MPa FR = 1.05 × 10^−4^ kg/s	[82,83]

S: Solvent; T: temperature; P: pressure; FR: flow rate; PS: particle size; S/RM: solvent/raw material; SE: static extraction; DE: dynamic extraction.

**Table 2:** Bioactive compounds and their application as ingredients in the formulation of healthy foods and their preventive effect on health.

Food Matrices and Residues	Bioactive Compounds	Applications	Effects	References
Vegetables and fruits				
Asparagus (*Asparagus officinalis* L. and *Asparagus racemosus*)	Phenolic compounds (APM) Phenolic acids (3-*O*-feruloylquinic acid)	Cookies and pharmaceutical products	-Antimicrobial, antiulcer, anti-diarrhoeal, and antioxidant activity -Neuroprotective and antipyretic effect	[92,93,94]
Spinach (*Spinacia oleracea* L.)	Polyphenols Phenolic compounds Carotenoids	Snacks and powders	Antioxidant activity	[95,96,97]
Parsley and seeds (*Petroselinum* *crispum*)	Phenolic compounds (Apin and Malonyl-apin)	Fortification wheat pasta and omelets.	-Protective effect against lipid and cholesterol Oxidation -Antiproliferative effect on carcinoma cells and antioxidant activity	[98,99,100]
Blackberries and leaves (*Morus nigra* L.)	Anthocyanins	Tea beverages	-Excellent antiadipogenic and inhibitory activity of lipid accumulation -Good cytotoxic and anti-inflammatory activity -Analgesic for symptoms of premenstrual tension as an infusion or decoction	[101]
Goji berry (*Lycium barbarum* L.)	Flavonols Phenolic acids	Tea beverages, juices, powders and dairy products	-Prebiotic effect -Anticarcinogenic, antioxidant, anti-inflammatory and anti-neurodegenerative effect -Reduce de risk of ocular, nephrological, and liver diseases.	[102,103,104]
Juçara and its residues (*Euterpe edulis* Mart.)	Anthocyanins	Uses as meat and poultry additives, beverages, and powders	-Antibiotic effect -Obesity and weight control -Anti-inflammatory effect	[105,106,107]
Herbs or species				
Rosemary (*Rosmarinus officinalis* L.)	Phenolic diterpenes (carnosol, scutellarein) Flavonoids (genkwanin)	Food coating materials, tea beverages, powders, additive for meat and fish products	-Antioxidant, antitumor, and anti-inflammatory activity	[30,108,109]
Lemon balm (*Melissa officinalis*)	Rosmarinic acid and its derivatives	Uses as additive in bakery and chocolate products	-Potential to prevent and manage mental, gastrointestinal disorders, and sleep disturbance -Antioxidant and antimicrobial properties	[110,111]
Turmeric (*Curcuma* *longa L.*)	Curcumin	Pickles, sauces, and mixtures with mustard	-Potential to prevent and manage noncommunicable diseases related to oxidative stress, anti-inflammatory, carminative, antiseptic, and antioxidant properties	[112]
Microalgae or macroalgae				
Spirulina microalga: (*Spirulina platensis*)	Carotenoids (zeaxanthin and β-carotene) Phenolic compounds Chlorophyll	Fortified yogurt	-Antioxidant, anti-inflammatory, and antibacterial activity -Good action of active ingredients in specific sites of the human body	[113]
Onion peel (*Allium cepa*)	Flavonols (quercetin)	Tea beverages, application in food package	-Cardioprotective and neuroprotective effect. -Antiobesity, antioxidant, antidiabetic, anticancer and antimicrobial activity.	[114,115,116]
Orange peel (*Citrus x sinensis*)	Flavonoids Glycosylated (hesperidin)	-Uses as supplementary ingredient in functional food products such as: beverages, dairy, and extruded products -Coloring and flavoring agent for food products	-Antioxidant and antimicrobial activity	[117]
Pomegranate peel (*Punica granatum* L.)	Punicalagin	Food coating, additive for date bars	-Antioxidant activity	[118,119]
Avocado peel (*Persea americana* Mill.)	Procyanidins Flavonols Hydroxybenzoic and hydroxycinnamic acids	Special culinary oil	-Used to treat hypercholesterolemia, hypertension, diabetes, and fatty liver disease -Reduces cardiometabolic risk and has anticancer and antimicrobial properties	[70]
Mango peel (*Mangifera indica* L.)	Phenolic compounds	Fortification of dairy and bakery products	-Antioxidant activity -Glycemic index control	[120,121]
Seeds				
Avocado seed (*Persea americana*)	Phenolic compounds Condensed tannins Phenolic acids Flavonoids	Food additive in meat products	-Antioxidant and antimicrobial activity	[122,123]
Grape seed (*Vitis* *vinifera*)	Catechins Proanthocyanidins	Special oils	-Strong antioxidants to combat oxidative stress -Inhibit tyrosinase activity -Prevent skin disorders due to hyperpigmentation	[72]
Bagasse				
Blueberry bagasse (*Vaccinium myrtillus* L.)	Anthocyanins	Food supplements	-Consumption protects against the development of cardiovascular diseases, diabetes, cancer, and neurodegenerative diseases -Powerful antioxidant activity and ability to modulate signaling pathways	[124]

## Data Availability

All the relevant data used for the paper are in the text or the cited references.
